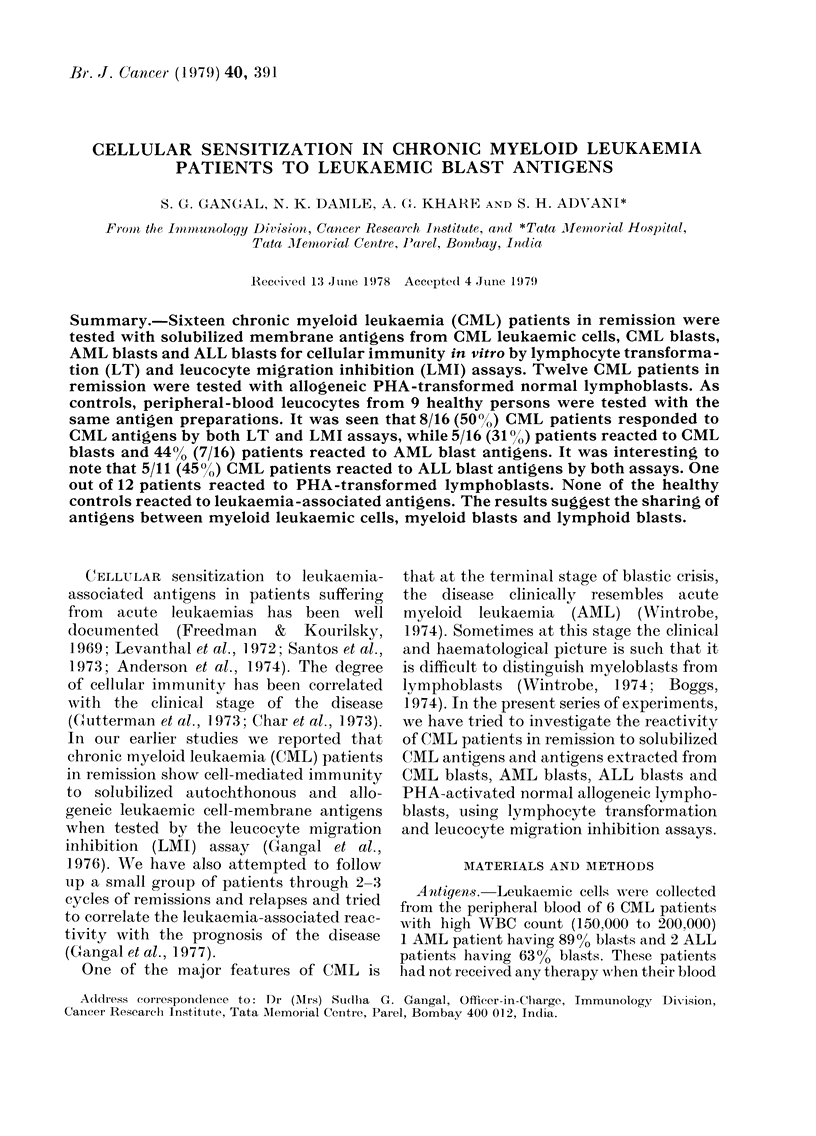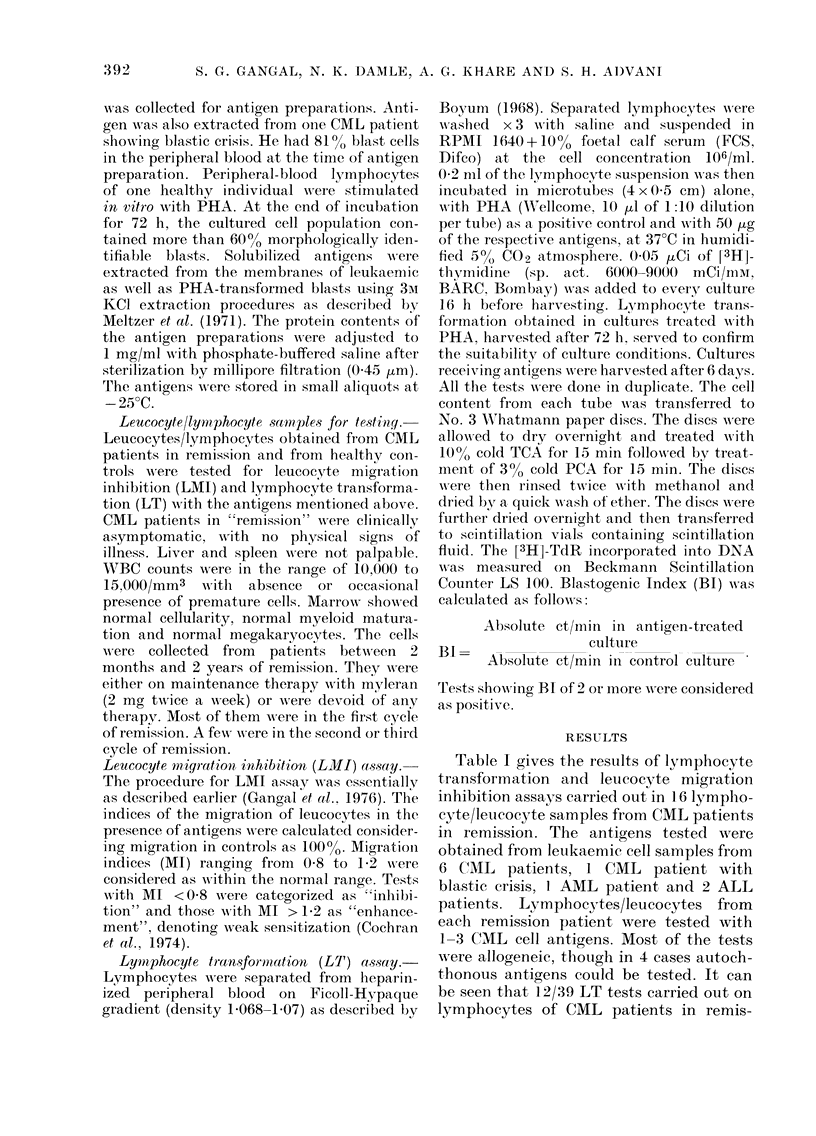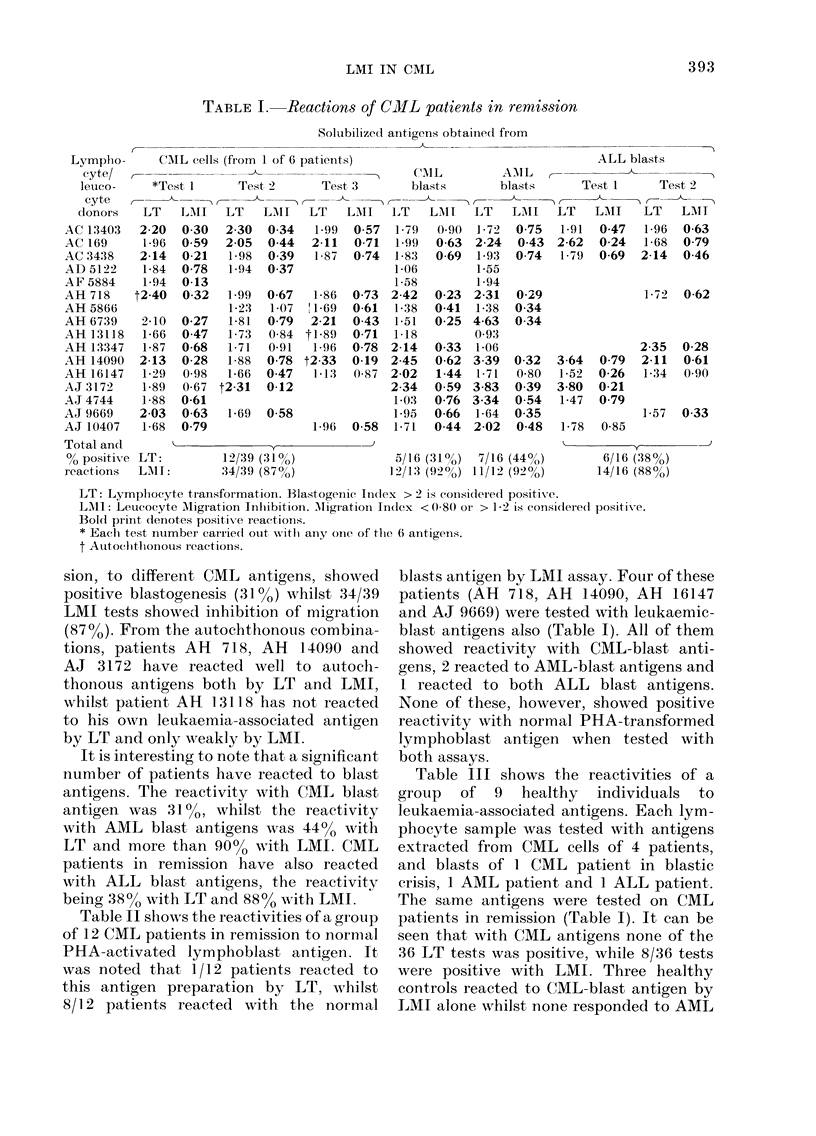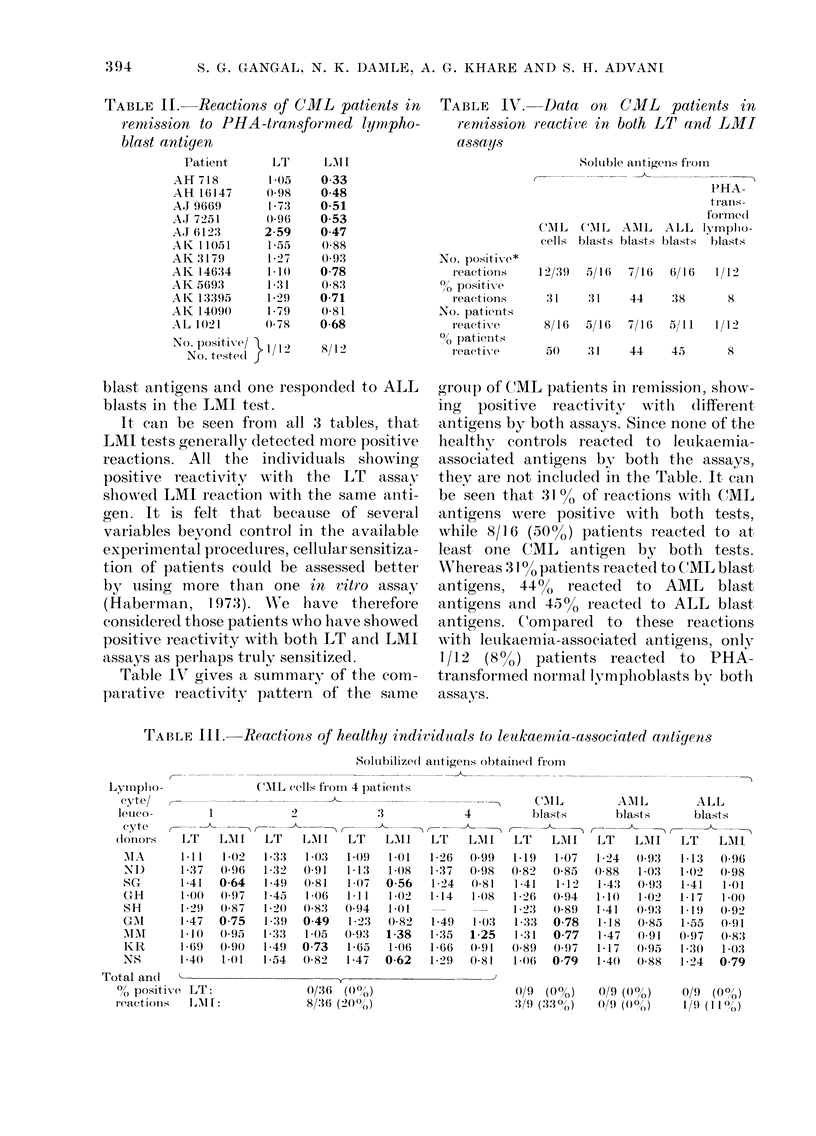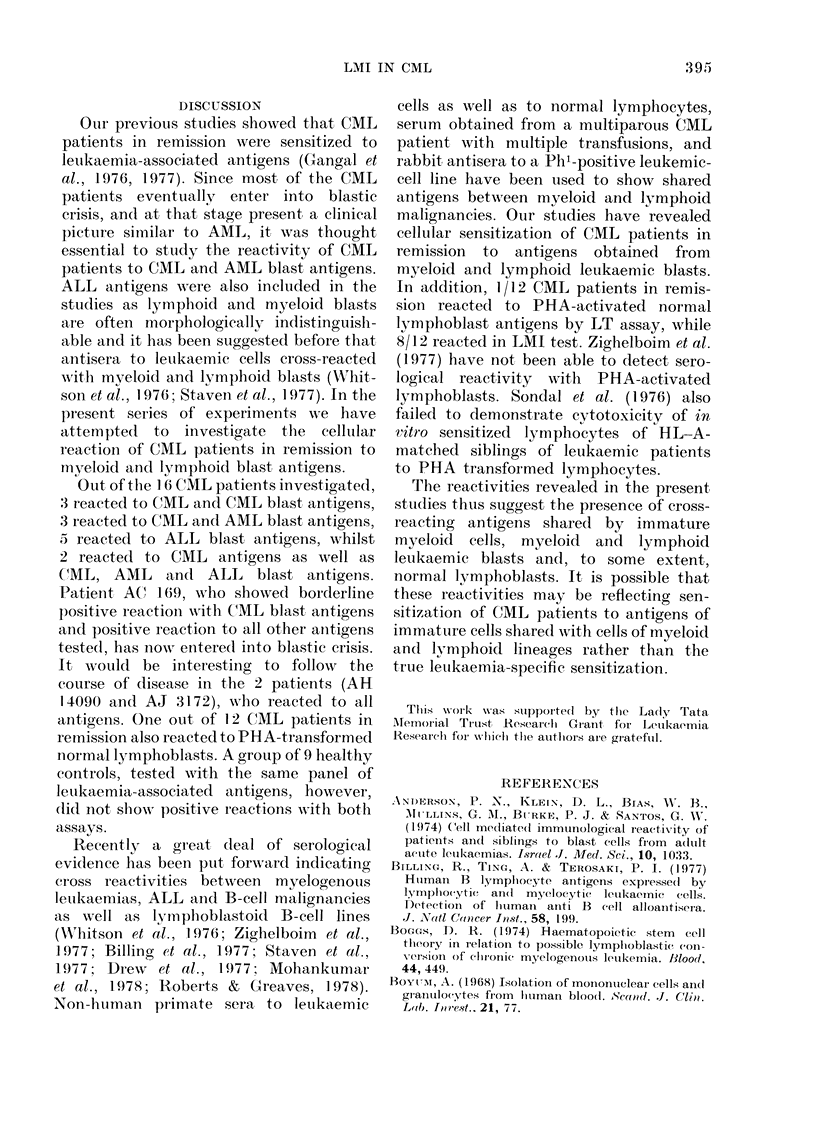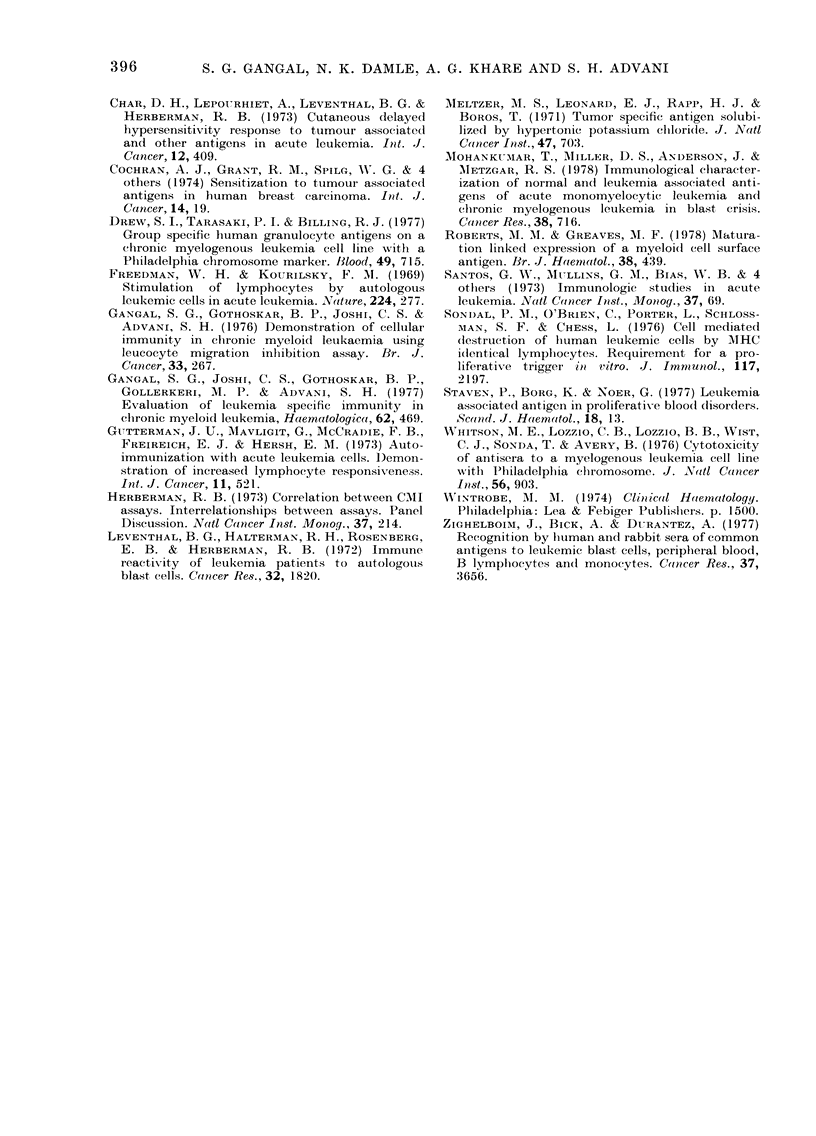# Cellular sensitization in chronic myeloid leukaemia patients to leukaemic blast antigens.

**DOI:** 10.1038/bjc.1979.193

**Published:** 1979-09

**Authors:** S. G. Gangal, N. K. Damle, A. G. Khare, S. H. Advani

## Abstract

Sixteen chronic myeloid leukaemia (CML) patients in remission were tested with solubilized membrane antigens from CML leukaemic cells, CML blasts, AML blasts and ALL blasts for cellular immunity in vitro by lymphocyte transformation (LT) and leucocyte migration inhibition (LMI) assays. Twelve CML patients in remission were tested with allogeneic PHA-transformed normal lymphoblasts. As controls, peripheral-blood leucocytes from 9 healthy persons were tested with the same antigen preparations. It was seen that 8/16 (50%) CML patients responded to CML antigens by both LT and LMI assays, while 5/16 (31%) patients reacted to CML blasts and 44% (7/16) patients reacted to AML blast antigens. It was interesting to note that 5/11 (45%) CML patients reacted to ALL blast antigens by both assays. One out of 12 patients reacted to PHA-transformed lymphoblasts. None of the healthy controls reacted to leukaemia-associated antigens. The results suggest the sharing of antigens between myeloid leukaemic cells, myeloid blasts and lymphoid blasts.


*Br. J. Cancer* (1979) **40**, 391

### CELLULAR SENSITIZATION IN CHRONIC MYELOID LEUKAEMIA PATIENTS TO LEUKAEMIC BLAST ANTIGENS

S. G. GANGAL, N. K. DAMLE, A. G. KHARE AND S. H. ADVANI*

*From the Immunology Division, Cancer Research Institute, and *Tata Memorial Hospital, Tata Memorial Centre, Parel, Bombay, India*

Received 13 June 1978 Accepted 4 June 1979

**Summary.—Sixteen chronic myeloid leukaemia (CML) patients in remission were tested with solubilized membrane antigens from CML leukaemic cells, CML blasts, AML blasts and ALL blasts for cellular immunity *in vitro* by lymphocyte transformation (LT) and leucocyte migration inhibition (LMI) assays. Twelve CML patients in remission were tested with allogeneic PHA-transformed normal lymphoblasts. As controls, peripheral-blood leucocytes from 9 healthy persons were tested with the same antigen preparations. It was seen that 8/16 (50%) CML patients responded to CML antigens by both LT and LMI assays, while 5/16 (31%) patients reacted to CML blasts and 44% (7/16) patients reacted to AML blast antigens. It was interesting to note that 5/11 (45%) CML patients reacted to ALL blast antigens by both assays. One out of 12 patients reacted to PHA-transformed lymphoblasts. None of the healthy controls reacted to leukaemia-associated antigens. The results suggest the sharing of antigens between myeloid leukaemic cells, myeloid blasts and lymphoid blasts.**

CELLULAR sensitization to leukaemia-associated antigens in patients suffering from acute leukaemias has been well documented (Freedman & Kourilsky, 1969; Levanthal *et al.*, 1972; Santos *et al.*, 1973; Anderson *et al.*, 1974). The degree of cellular immunity has been correlated with the clinical stage of the disease (Gutterman *et al.*, 1973; Char *et al.*, 1973). In our earlier studies we reported that chronic myeloid leukaemia (CML) patients in remission show cell-mediated immunity to solubilized autochthonous and allogeneic leukaemic cell-membrane antigens when tested by the leucocyte migration inhibition (LMI) assay (Gangal *et al.*, 1976). We have also attempted to follow up a small group of patients through 2–3 cycles of remissions and relapses and tried to correlate the leukaemia-associated reactivity with the prognosis of the disease (Gangal *et al.*, 1977).

One of the major features of CML is that at the terminal stage of blastic crisis, the disease clinically resembles acute myeloid leukaemia (AML) (Wintrobe, 1974). Sometimes at this stage the clinical and haematological picture is such that it is difficult to distinguish myeloblasts from lymphoblasts (Wintrobe, 1974; Boggs, 1974). In the present series of experiments, we have tried to investigate the reactivity of CML patients in remission to solubilized CML antigens and antigens extracted from CML blasts, AML blasts, ALL blasts and PHA-activated normal allogeneic lymphoblasts, using lymphocyte transformation and leucocyte migration inhibition assays.

### MATERIALS AND METHODS

*Antigens.*—Leukaemic cells were collected from the peripheral blood of 6 CML patients with high WBC count (150,000 to 200,000) 1 AML patient having 89% blasts and 2 ALL patients having 63% blasts. These patients had not received any therapy when their blood

Address correspondence to: Dr (Mrs) Sudha G. Gangal, Officer-in-Charge, Immunology Division, Cancer Research Institute, Tata Memorial Centre, Parel, Bombay 400 012, India.

was collected for antigen preparations. Antigen was also extracted from one CML patient showing blastic crisis. He had 81% blast cells in the peripheral blood at the time of antigen preparation. Peripheral-blood lymphocytes of one healthy individual were stimulated *in vitro* with PHA. At the end of incubation for 72 h, the cultured cell population contained more than 60% morphologically identifiable blasts. Solubilized antigens were extracted from the membranes of leukaemic as well as PHA-transformed blasts using 3M KCl extraction procedures as described by Meltzer *et al.* (1971). The protein contents of the antigen preparations were adjusted to 1 mg/ml with phosphate-buffered saline after sterilization by millipore filtration (0·45 $\mu$m). The antigens were stored in small aliquots at −25°C.

*Leucocyte/lymphocyte samples for testing.*—Leucocytes/lymphocytes obtained from CML patients in remission and from healthy controls were tested for leucocyte migration inhibition (LMI) and lymphocyte transformation (LT) with the antigens mentioned above. CML patients in "remission" were clinically asymptomatic, with no physical signs of illness. Liver and spleen were not palpable. WBC counts were in the range of 10,000 to 15,000/mm$^3$ with absence or occasional presence of premature cells. Marrow showed normal cellularity, normal myeloid maturation and normal megakaryocytes. The cells were collected from patients between 2 months and 2 years of remission. They were either on maintenance therapy with myleran (2 mg twice a week) or were devoid of any therapy. Most of them were in the first cycle of remission. A few were in the second or third cycle of remission.

*Leucocyte migration inhibition (LMI) assay.*—The procedure for LMI assay was essentially as described earlier (Gangal *et al.*, 1976). The indices of the migration of leucocytes in the presence of antigens were calculated considering migration in controls as 100%. Migration indices (MI) ranging from 0·8 to 1·2 were considered as within the normal range. Tests with MI <0·8 were categorized as "inhibition" and those with MI >1·2 as "enhancement", denoting weak sensitization (Cochran *et al.*, 1974).

*Lymphocyte transformation (LT) assay.*—Lymphocytes were separated from heparinized peripheral blood on Ficoll-Hypaque gradient (density 1·068–1·07) as described by Boyum (1968). Separated lymphocytes were washed ×3 with saline and suspended in RPMI 1640+10% foetal calf serum (FCS, Difco) at the cell concentration 10$^6$/ml. 0·2 ml of the lymphocyte suspension was then incubated in microtubes (4×0·5 cm) alone, with PHA (Wellcome, 10 $\mu$l of 1:10 dilution per tube) as a positive control and with 50 $\mu$g of the respective antigens, at 37°C in humidified 5% $CO_2$ atmosphere. 0·05 $\mu$Ci of [$^3$H]-thymidine (sp. act. 6000–9000 mCi/mM, BARC, Bombay) was added to every culture 16 h before harvesting. Lymphocyte transformation obtained in cultures treated with PHA, harvested after 72 h, served to confirm the suitability of culture conditions. Cultures receiving antigens were harvested after 6 days. All the tests were done in duplicate. The cell content from each tube was transferred to No. 3 Whatmann paper discs. The discs were allowed to dry overnight and treated with 10% cold TCA for 15 min followed by treatment of 3% cold PCA for 15 min. The discs were then rinsed twice with methanol and dried by a quick wash of ether. The discs were further dried overnight and then transferred to scintillation vials containing scintillation fluid. The [$^3$H]-TdR incorporated into DNA was measured on Beckmann Scintillation Counter LS 100. Blastogenic Index (BI) was calculated as follows:

$$BI = \frac{\text{Absolute ct/min in antigen-treated culture}}{\text{Absolute ct/min in control culture}}.$$

Tests showing BI of 2 or more were considered as positive.

### RESULTS

Table I gives the results of lymphocyte transformation and leucocyte migration inhibition assays carried out in 16 lymphocyte/leucocyte samples from CML patients in remission. The antigens tested were obtained from leukaemic cell samples from 6 CML patients, 1 CML patient with blastic crisis, 1 AML patient and 2 ALL patients. Lymphocytes/leucocytes from each remission patient were tested with 1–3 CML cell antigens. Most of the tests were allogeneic, though in 4 cases autochthonous antigens could be tested. It can be seen that 12/39 LT tests carried out on lymphocytes of CML patients in remis-

TABLE I.—*Reactions of CML patients in remission*

| Lymphocyte/leucocyte donors | Solubilized antigens obtained from: CML cells (from 1 of 6 patients) *Test 1 LT | *Test 1 LMI | Test 2 LT | Test 2 LMI | Test 3 LT | Test 3 LMI | CML blasts LT | CML blasts LMI | AML blasts LT | AML blasts LMI | ALL blasts Test 1 LT | ALL blasts Test 1 LMI | ALL blasts Test 2 LT | ALL blasts Test 2 LMI |
|---|---|---|---|---|---|---|---|---|---|---|---|---|---|---|
| AC 13403 | **2·20** | **0·30** | **2·30** | **0·34** | 1·99 | **0·57** | 1·79 | 0·90 | 1·72 | **0·75** | 1·91 | **0·47** | 1·96 | **0·63** |
| AC 169 | 1·96 | **0·59** | **2·05** | **0·44** | **2·11** | **0·71** | 1·99 | **0·63** | **2·24** | **0·43** | **2·62** | **0·24** | 1·68 | **0·79** |
| AC 3438 | **2·14** | **0·21** | 1·98 | **0·39** | 1·87 | **0·74** | 1·83 | **0·69** | 1·93 | **0·74** | 1·79 | **0·69** | **2·14** | **0·46** |
| AD 5122 | 1·84 | **0·78** | 1·94 | **0·37** | | | 1·06 | | 1·55 | | | | | |
| AF 5884 | 1·94 | **0·13** | | | | | 1·58 | | 1·94 | | | | | |
| AH 718 | †**2·40** | **0·32** | 1·99 | **0·67** | 1·86 | **0·73** | **2·42** | **0·23** | **2·31** | **0·29** | | | 1·72 | **0·62** |
| AH 5866 | | | 1·23 | 1·07 | †1·69 | **0·61** | 1·38 | **0·41** | 1·38 | **0·34** | | | | |
| AH 6739 | 2·10 | **0·27** | 1·81 | **0·79** | **2·21** | **0·43** | 1·51 | **0·25** | **4·63** | **0·34** | | | | |
| AH 13118 | 1·66 | **0·47** | 1·73 | 0·84 | †1·89 | **0·71** | 1·18 | | 0·93 | | | | | |
| AH 13347 | 1·87 | **0·68** | 1·71 | 0·91 | 1·96 | **0·78** | **2·14** | **0·33** | 1·06 | | | | **2·35** | **0·28** |
| AH 14090 | **2·13** | **0·28** | 1·88 | **0·78** | †**2·33** | **0·19** | **2·45** | **0·62** | **3·39** | **0·32** | **3·64** | **0·79** | **2·11** | **0·61** |
| AH 16147 | 1·29 | 0·98 | 1·66 | **0·47** | 1·13 | 0·87 | **2·02** | **1·44** | 1·71 | 0·80 | 1·52 | **0·26** | 1·34 | 0·90 |
| AJ 3172 | 1·89 | 0·67 | †**2·31** | **0·12** | | | **2·34** | **0·59** | **3·83** | **0·39** | **3·80** | **0·21** | | |
| AJ 4744 | 1·88 | **0·61** | | | | | 1·03 | **0·76** | **3·34** | **0·54** | 1·47 | **0·79** | | |
| AJ 9669 | **2·03** | **0·63** | 1·69 | **0·58** | | | 1·95 | **0·66** | 1·64 | **0·35** | | | 1·57 | **0·33** |
| AJ 10407 | 1·68 | **0·79** | | | 1·96 | **0·58** | 1·71 | **0·44** | **2·02** | **0·48** | 1·78 | 0·85 | | |
| Total and % positive reactions LT: | 12/39 (31%) | | | | | | 5/16 (31%) | | 7/16 (44%) | | 6/16 (38%) | | | |
| LMI: | 34/39 (87%) | | | | | | 12/13 (92%) | | 11/12 (92%) | | 14/16 (88%) | | | |

LT: Lymphocyte transformation. Blastogenic Index > 2 is considered positive.
LMI: Leucocyte Migration Inhibition. Migration Index < 0·80 or > 1·2 is considered positive.
Bold print denotes positive reactions.
* Each test number carried out with any one of the 6 antigens.
† Autochthonous reactions.

sion, to different CML antigens, showed positive blastogenesis (31%) whilst 34/39 LMI tests showed inhibition of migration (87%). From the autochthonous combinations, patients AH 718, AH 14090 and AJ 3172 have reacted well to autochthonous antigens both by LT and LMI, whilst patient AH 13118 has not reacted to his own leukaemia-associated antigen by LT and only weakly by LMI.

It is interesting to note that a significant number of patients have reacted to blast antigens. The reactivity with CML blast antigen was 31%, whilst the reactivity with AML blast antigens was 44% with LT and more than 90% with LMI. CML patients in remission have also reacted with ALL blast antigens, the reactivity being 38% with LT and 88% with LMI.

Table II shows the reactivities of a group of 12 CML patients in remission to normal PHA-activated lymphoblast antigen. It was noted that 1/12 patients reacted to this antigen preparation by LT, whilst 8/12 patients reacted with the normal blasts antigen by LMI assay. Four of these patients (AH 718, AH 14090, AH 16147 and AJ 9669) were tested with leukaemic-blast antigens also (Table I). All of them showed reactivity with CML-blast antigens, 2 reacted to AML-blast antigens and 1 reacted to both ALL blast antigens. None of these, however, showed positive reactivity with normal PHA-transformed lymphoblast antigen when tested with both assays.

Table III shows the reactivities of a group of 9 healthy individuals to leukaemia-associated antigens. Each lymphocyte sample was tested with antigens extracted from CML cells of 4 patients, and blasts of 1 CML patient in blastic crisis, 1 AML patient and 1 ALL patient. The same antigens were tested on CML patients in remission (Table I). It can be seen that with CML antigens none of the 36 LT tests was positive, while 8/36 tests were positive with LMI. Three healthy controls reacted to CML-blast antigen by LMI alone whilst none responded to AML

TABLE II.—*Reactions of CML patients in remission to PHA-transformed lymphoblast antigen*

| Patient | LT | LMI |
|---|---|---|
| AH 718 | 1·05 | **0·33** |
| AH 16147 | 0·98 | **0·48** |
| AJ 9669 | 1·73 | **0·51** |
| AJ 7251 | 0·96 | **0·53** |
| AJ 6123 | **2·59** | **0·47** |
| AK 11051 | 1·55 | 0·88 |
| AK 3179 | 1·27 | 0·93 |
| AK 14634 | 1·10 | **0·78** |
| AK 5693 | 1·31 | 0·83 |
| AK 13395 | 1·29 | **0·71** |
| AK 14090 | 1·79 | 0·81 |
| AL 1021 | 0·78 | **0·68** |
| No. positive/ No. tested | 1/12 | 8/12 |

TABLE IV.—*Data on CML patients in remission reactive in both LT and LMI assays*

| | Soluble antigens from | | | | |
|---|---|---|---|---|---|
| | CML cells | CML blasts | AML blasts | ALL blasts | PHA-transformed lymphoblasts |
| No. positive* reactions | 12/39 | 5/16 | 7/16 | 6/16 | 1/12 |
| % positive reactions | 31 | 31 | 44 | 38 | 8 |
| No. patients reactive | 8/16 | 5/16 | 7/16 | 5/11 | 1/12 |
| % patients reactive | 50 | 31 | 44 | 45 | 8 |

blast antigens and one responded to ALL blasts in the LMI test.

It can be seen from all 3 tables, that LMI tests generally detected more positive reactions. All the individuals showing positive reactivity with the LT assay showed LMI reaction with the same antigen. It is felt that because of several variables beyond control in the available experimental procedures, cellular sensitization of patients could be assessed better by using more than one *in vitro* assay (Haberman, 1973). We have therefore considered those patients who have showed positive reactivity with both LT and LMI assays as perhaps truly sensitized.

Table IV gives a summary of the comparative reactivity pattern of the same group of CML patients in remission, showing positive reactivity with different antigens by both assays. Since none of the healthy controls reacted to leukaemia-associated antigens by both the assays, they are not included in the Table. It can be seen that 31% of reactions with CML antigens were positive with both tests, while 8/16 (50%) patients reacted to at least one CML antigen by both tests. Whereas 31% patients reacted to CML blast antigens, 44% reacted to AML blast antigens and 45% reacted to ALL blast antigens. Compared to these reactions with leukaemia-associated antigens, only 1/12 (8%) patients reacted to PHA-transformed normal lymphoblasts by both assays.

TABLE III.—*Reactions of healthy individuals to leukaemia-associated antigens*

| Lymphocyte/ leucocyte donors | Solubilized antigens obtained from: CML cells from 4 patients: 1 LT | 1 LMI | 2 LT | 2 LMI | 3 LT | 3 LMI | 4 LT | 4 LMI | CML blasts LT | CML blasts LMI | AML blasts LT | AML blasts LMI | ALL blasts LT | ALL blasts LMI |
|---|---|---|---|---|---|---|---|---|---|---|---|---|---|---|
| MA | 1·11 | 1·02 | 1·33 | 1·03 | 1·09 | 1·01 | 1·26 | 0·99 | 1·19 | 1·07 | 1·24 | 0·93 | 1·13 | 0·96 |
| ND | 1·37 | 0·96 | 1·32 | 0·91 | 1·13 | 1·08 | 1·37 | 0·98 | 0·82 | 0·85 | 0·88 | 1·03 | 1·02 | 0·98 |
| SG | 1·41 | **0·64** | 1·49 | 0·81 | 1·07 | **0·56** | 1·24 | 0·81 | 1·41 | 1·12 | 1·43 | 0·93 | 1·41 | 1·01 |
| GH | 1·00 | 0·97 | 1·45 | 1·06 | 1·11 | 1·02 | 1·14 | 1·08 | 1·26 | 0·94 | 1·10 | 1·02 | 1·17 | 1·00 |
| SH | 1·29 | 0·87 | 1·20 | 0·83 | 0·94 | 1·01 | — | — | 1·23 | 0·89 | 1·41 | 0·93 | 1·19 | 0·92 |
| GM | 1·47 | **0·75** | 1·39 | **0·49** | 1·23 | 0·82 | 1·49 | 1·03 | 1·33 | **0·78** | 1·18 | 0·85 | 1·55 | 0·91 |
| MM | 1·10 | 0·95 | 1·33 | 1·05 | 0·93 | **1·38** | 1·35 | **1·25** | 1·31 | **0·77** | 1·47 | 0·91 | 0·97 | 0·83 |
| KR | 1·69 | 0·90 | 1·49 | **0·73** | 1·65 | 1·06 | 1·66 | 0·91 | 0·89 | 0·97 | 1·17 | 0·95 | 1·30 | 1·03 |
| NS | 1·40 | 1·01 | 1·54 | 0·82 | 1·47 | **0·62** | 1·29 | 0·81 | 1·06 | **0·79** | 1·40 | 0·88 | 1·24 | **0·79** |
| Total and % positive reactions LT: | 0/36 (0%) | | | | | | | | 0/9 (0%) | | 0/9 (0%) | | 0/9 (0%) | |
| Total and % positive reactions LMI: | 8/36 (20%) | | | | | | | | 3/9 (33%) | | 0/9 (0%) | | 1/9 (11%) | |

### DISCUSSION

Our previous studies showed that CML patients in remission were sensitized to leukaemia-associated antigens (Gangal *et al.*, 1976, 1977). Since most of the CML patients eventually enter into blastic crisis, and at that stage present a clinical picture similar to AML, it was thought essential to study the reactivity of CML patients to CML and AML blast antigens. ALL antigens were also included in the studies as lymphoid and myeloid blasts are often morphologically indistinguishable and it has been suggested before that antisera to leukaemic cells cross-reacted with myeloid and lymphoid blasts (Whitson *et al.*, 1976; Staven *et al.*, 1977). In the present series of experiments we have attempted to investigate the cellular reaction of CML patients in remission to myeloid and lymphoid blast antigens.

Out of the 16 CML patients investigated, 3 reacted to CML and CML blast antigens, 3 reacted to CML and AML blast antigens, 5 reacted to ALL blast antigens, whilst 2 reacted to CML antigens as well as CML, AML and ALL blast antigens. Patient AC 169, who showed borderline positive reaction with CML blast antigens and positive reaction to all other antigens tested, has now entered into blastic crisis. It would be interesting to follow the course of disease in the 2 patients (AH 14090 and AJ 3172), who reacted to all antigens. One out of 12 CML patients in remission also reacted to PHA-transformed normal lymphoblasts. A group of 9 healthy controls, tested with the same panel of leukaemia-associated antigens, however, did not show positive reactions with both assays.

Recently a great deal of serological evidence has been put forward indicating cross reactivities between myelogenous leukaemias, ALL and B-cell malignancies as well as lymphoblastoid B-cell lines (Whitson *et al.*, 1976; Zighelboim *et al.*, 1977; Billing *et al.*, 1977; Staven *et al.*, 1977; Drew *et al.*, 1977; Mohankumar *et al.*, 1978; Roberts & Greaves, 1978). Non-human primate sera to leukaemic cells as well as to normal lymphocytes, serum obtained from a multiparous CML patient with multiple transfusions, and rabbit antisera to a $Ph^1$-positive leukemic-cell line have been used to show shared antigens between myeloid and lymphoid malignancies. Our studies have revealed cellular sensitization of CML patients in remission to antigens obtained from myeloid and lymphoid leukaemic blasts. In addition, 1/12 CML patients in remission reacted to PHA-activated normal lymphoblast antigens by LT assay, while 8/12 reacted in LMI test. Zighelboim *et al.* (1977) have not been able to detect serological reactivity with PHA-activated lymphoblasts. Sondal *et al.* (1976) also failed to demonstrate cytotoxicity of *in vitro* sensitized lymphocytes of HL-A-matched siblings of leukaemic patients to PHA transformed lymphocytes.

The reactivities revealed in the present studies thus suggest the presence of cross-reacting antigens shared by immature myeloid cells, myeloid and lymphoid leukaemic blasts and, to some extent, normal lymphoblasts. It is possible that these reactivities may be reflecting sensitization of CML patients to antigens of immature cells shared with cells of myeloid and lymphoid lineages rather than the true leukaemia-specific sensitization.

This work was supported by the Lady Tata Memorial Trust Research Grant for Leukaemia Research for which the authors are grateful.

### REFERENCES

ANDERSON, P. N., KLEIN, D. L., BIAS, W. B., MULLINS, G. M., BURKE, P. J. & SANTOS, G. W. (1974) Cell mediated immunological reactivity of patients and siblings to blast cells from adult acute leukaemias. *Israel J. Med. Sci.*, **10**, 1033.

BILLING, R., TING, A. & TEROSAKI, P. I. (1977) Human B lymphocyte antigens expressed by lymphocytic and myelocytic leukaemic cells. Detection of human anti B cell alloantisera. *J. Natl Cancer Inst.*, **58**, 199.

BOGGS, D. R. (1974) Haematopoietic stem cell theory in relation to possible lymphoblastic conversion of chronic myelogenous leukemia. *Blood*, **44**, 449.

BOYUM, A. (1968) Isolation of mononuclear cells and granulocytes from human blood. *Scand. J. Clin. Lab. Invest.*, **21**, 77.

CHAR, D. H., LEPOURHIET, A., LEVENTHAL, B. G. & HERBERMAN, R. B. (1973) Cutaneous delayed hypersensitivity response to tumour associated and other antigens in acute leukemia. *Int. J. Cancer*, **12**, 409.

COCHRAN, A. J., GRANT, R. M., SPILG, W. G. & 4 others (1974) Sensitization to tumour associated antigens in human breast carcinoma. *Int. J. Cancer*, **14**, 19.

DREW, S. I., TARASAKI, P. I. & BILLING, R. J. (1977) Group specific human granulocyte antigens on a chronic myelogenous leukemia cell line with a Philadelphia chromosome marker. *Blood*, **49**, 715.

FREEDMAN, W. H. & KOURILSKY, F. M. (1969) Stimulation of lymphocytes by autologous leukemic cells in acute leukemia. *Nature*, **224**, 277.

GANGAL, S. G., GOTHOSKAR, B. P., JOSHI, C. S. & ADVANI, S. H. (1976) Demonstration of cellular immunity in chronic myeloid leukaemia using leucocyte migration inhibition assay. *Br. J. Cancer*, **33**, 267.

GANGAL, S. G., JOSHI, C. S., GOTHOSKAR, B. P., GOLLERKERI, M. P. & ADVANI, S. H. (1977) Evaluation of leukemia specific immunity in chronic myeloid leukemia, *Haematologica*, **62**, 469.

GUTTERMAN, J. U., MAVLIGIT, G., MCCRADIE, F. B., FREIREICH, E. J. & HERSH, E. M. (1973) Auto-immunization with acute leukemia cells. Demonstration of increased lymphocyte responsiveness. *Int. J. Cancer*, **11**, 521.

HERBERMAN, R. B. (1973) Correlation between CMI assays. Interrelationships between assays. Panel Discussion. *Natl Cancer Inst. Monog.*, **37**, 214.

LEVENTHAL, B. G., HALTERMAN, R. H., ROSENBERG, E. B. & HERBERMAN, R. B. (1972) Immune reactivity of leukemia patients to autologous blast cells. *Cancer Res.*, **32**, 1820.

MELTZER, M. S., LEONARD, E. J., RAPP, H. J. & BOROS, T. (1971) Tumor specific antigen solubilized by hypertonic potassium chloride. *J. Natl Cancer Inst.*, **47**, 703.

MOHANKUMAR, T., MILLER, D. S., ANDERSON, J. & METZGAR, R. S. (1978) Immunological characterization of normal and leukemia associated antigens of acute monomyelocytic leukemia and chronic myelogenous leukemia in blast crisis. *Cancer Res.*, **38**, 716.

ROBERTS, M. M. & GREAVES, M. F. (1978) Maturation linked expression of a myeloid cell surface antigen. *Br. J. Haematol.*, **38**, 439.

SANTOS, G. W., MULLINS, G. M., BIAS, W. B. & 4 others (1973) Immunologic studies in acute leukemia. *Natl Cancer Inst., Monog.*, **37**, 69.

SONDAL, P. M., O'BRIEN, C., PORTER, L., SCHLOSSMAN, S. F. & CHESS, L. (1976) Cell mediated destruction of human leukemic cells by MHC identical lymphocytes. Requirement for a proliferative trigger *in vitro*. *J. Immunol.*, **117**, 2197.

STAVEN, P., BORG, K. & NOER, G. (1977) Leukemia associated antigen in proliferative blood disorders. *Scand. J. Haematol.*, **18**, 13.

WHITSON, M. E., LOZZIO, C. B., LOZZIO, B. B., WIST, C. J., SONDA, T. & AVERY, B. (1976) Cytotoxicity of antisera to a myelogenous leukemia cell line with Philadelphia chromosome. *J. Natl Cancer Inst.*, **56**, 903.

WINTROBE, M. M. (1974) *Clinical Haematology*. Philadelphia: Lea & Febiger Publishers. p. 1500.

ZIGHELBOIM, J., BICK, A. & DURANTEZ, A. (1977) Recognition by human and rabbit sera of common antigens to leukemic blast cells, peripheral blood, B lymphocytes and monocytes. *Cancer Res.*, **37**, 3656.